# Global multi-method analysis of interaction parameters for reversibly self-associating macromolecules at high concentrations

**DOI:** 10.1038/s41598-021-84946-8

**Published:** 2021-03-11

**Authors:** Arun Parupudi, Sumit K. Chaturvedi, Regina Adão, Robert W. Harkness, Sonia Dragulin-Otto, Lewis E. Kay, Reza Esfandiary, Huaying Zhao, Peter Schuck

**Affiliations:** 1 Department of Dosage Form Design and Development, Biopharmaceuticals R&D, AstraZeneca, Gaithersburg, MD 20878 USA; 2 Dynamics of Macromolecular Assembly Section, Laboratory of Cellular Imaging and Macromolecular Biophysics, National Institute of Biomedical Imaging and Bioengineering, National Institutes of Health, Bethesda, MD 20817 USA; 3 Departments of Molecular Genetics, Biochemistry, and Chemistry, University of Toronto, Toronto, ON M5S 1A8 Canada; 4 The Hospital for Sick Children Research Institute, Program in Molecular Medicine, Toronto, ON M5G 0A4 Canada

**Keywords:** Analytical biochemistry, Biophysical methods, Physical chemistry, Blood proteins, Statistical physics, thermodynamics and nonlinear dynamics, Characterization and analytical techniques

## Abstract

Weak macromolecular interactions assume a dominant role in the behavior of highly concentrated solutions, and are at the center of a variety of fields ranging from colloidal chemistry to cell biology, neurodegenerative diseases, and manufacturing of protein drugs. They are frequently measured in different biophysical techniques in the form of second virial coefficients, and nonideality coefficients of sedimentation and diffusion, which may be related mechanistically to macromolecular distance distributions in solution and interparticle potentials. A problem arises for proteins where reversible self-association often complicates the concentration-dependent behavior, such that grossly inconsistent coefficients are measured in experiments based on different techniques, confounding quantitative conclusions. Here we present a global multi-method analysis that synergistically bridges gaps in resolution and sensitivity of orthogonal techniques. We demonstrate the method with a panel of monoclonal antibodies exhibiting different degrees of self-association. We show how their concentration-dependent behavior, examined by static and dynamic light scattering and sedimentation velocity, can be jointly described in a self-consistent framework that separates nonideality coefficients from self-association properties, and thereby extends the quantitative interpretation of nonideality coefficients to probe dynamics in highly concentrated protein solutions.

## Introduction

The solution state of concentrated macromolecular solutions has emerged as an important and urgent question in diverse fields: For example, a challenge in the manufacturing of protein therapeutics is the development of formulations with concentrations on the order of 100 mg/mL of protein that are injectable, low-viscosity, and non-immunogenic with a predictably long shelf-life^1–4^. While nature has solved a similar problem for eye lens crystallins, which remain soluble for decades at hundreds of mg/mL in a state with only short-range order ensuring lens transparency, their aggregation and liquid–liquid phase transition causes cataracts which is a world-wide leading cause of blindness^5,6^. Protein aggregation has also been identified as a key mechanism in several neurodegenerative diseases^7^, some of which have been associated with cellular condensates driven by liquid–liquid phase transitions^8^. The latter mechanism is increasingly recognized as a key principle of cellular organization^9^, with consequences for pharmacokinetics^10^. Solubility, aggregation, crystallization, and phase transitions are phenomena driven by subtle intermolecular forces that become dominant at high concentrations.

Unfortunately, protein concentrations of 100 mg/mL exceed the range of most physical chemistry techniques. However, measurements of the second virial coefficient (*B*_2_) and the nonideality coefficients of sedimentation (*k*_S_) and diffusion (*k*_D_) describing ‘soft’ thermodynamic and hydrodynamic interactions in semi-dilute solutions have been well-established as important tools to glean information on the structure of highly concentrated protein solutions^11–15^. The nonideality/virial coefficients can be measured by a variety of techniques, and in practice frequently include small angle and static light scattering (SLS), dynamic light scattering (DLS), and sedimentation velocity analytical ultracentrifugation (SV). While each technique reports on the concentration-dependence of different observables, their relationship has been firmly established for suspensions of non-interacting hard spheres, as 2*B*_2_ = *k*_S_ + *k*_D_^16^. Furthermore, statistical fluid mechanics theory has dissected contributions from macromolecular volume exclusion and the effect of macromolecular distance distribution on hydrodynamic interactions, all of which contribute to different extent to each of the parameters^17^. Using silica particles as a model system for non-interacting hard spheres, and combining static and dynamic light scattering with sedimentation experiments, Kops-Werkhoven and Fijnaut have first confirmed the theoretically predicted values of *k*_S_ =  +6.55 × $\overline{v}$ and *k*_D_ =  +1.45 × $\overline{v}$ (where $\overline{v}$ is the partial-specific volume) and verified the predicted relationship 2*B*_2_ = *k*_S_ + *k*_D_^18^. Theory has further evaluated how altered molecular distance distributions due to attractive and/or repulsive inter-particle potentials modulate hydrodynamic interactions^19–21^.

A compounding complication for concentrated protein solutions is the fact that many, if not most, proteins can form transient complexes, which may involve well-defined surface epitopes that produce oligomeric species of significant lifetime (on the time-scale of molecular motion) and thereby alter overall thermodynamic and hydrodynamic solution behavior^22^. Since proteins are not merely colloidal particles, additional questions arise regarding molecular collision frequency, lifetime and population of oligomeric fractions, and the associated probabilities of macromolecular conformational changes producing more stable aggregates. These aspects are paramount, for example, in the development of formulations for protein therapeutics^3,4^. Thus, the analysis of thermodynamic and hydrodynamic nonideality of proteins exhibiting certain degrees of self-association has emerged as an important problem, both from a theoretical as well as practical perspective.

Currently, conflicting frameworks can be found in the literature for the calculation and interpretation of nonideality coefficients, chiefly differing in adopted definitions either excplicitly including different self-association states, or—in a purely operational view of concentration-dependent observables—excluding consideration of self-association. In part, different approaches are imposed by experimental concentration range and resolution. The validity of the relationship 2*B*_2_ = *k*_S_ + *k*_D_ has not been questioned, but unfortunately, where multiple techniques have been used, the experimentally measured parameter values have often been grossly inconsistent. This discrepancy is most apparent for the popular measurement of nonideality of diffusion by DLS, which results in strongly negative *k*_D_-values in the presence of self-association that are often in conflict with moderate virial coefficients measured by SLS and with results from SV. Although this problem is usually understood as a result of different definitions and resolution, it has often limited interpretation to semi-quantitative considerations.

The goal of the present work is to develop a method for determining nonideality coefficients that are quantitative and self-consistent across different techniques, and to dissect specific oligomeric complex formation in self-association processes from nonideality defined as resulting from volume exclusion and ‘soft’ long-range interactions causing non-uniform macromolecular distance distributions^23^. We test the method with a set of monoclonal antibodies with different degrees of self-association and nonideality, which have been previously used as benchmarks (Supplementary Table S1)^12,14,24^. As experimental foundation we use the classical combination of SLS, DLS, and SV, which all probe size, shape, and motion of macromolecules free in solution. For single noninteracting and ideal species SLS measures the molecular weight *M*, DLS the diffusion coefficient *D*, and SV the sedimentation coefficient *s*, which are fundamentally connected in the famous Svedberg relationship $M(1 - \overline{v}\rho ) = {{sRT} \mathord{\left/ {\vphantom {{sRT} D}} \right. \kern-\nulldelimiterspace} D}$. In the presence of self-association and nonideality, we analyze in theory and demonstrate in practice why conventional combination of separate applications of these methods can fail, indicating inaccurate results. To overcome this problem, we apply global multi-method analysis (GMMA), which we have previously developed to enhance the study of energetics in multi-protein complexes^25,26^. While the previous models were concerned with multi-component mixtures in the dilute solutions, we extend these strategies here to the characterization of nonideality at high concentrations, and arrive at a good description of all data with self-consistent quantitative results. Complementary to the description of nonideality, we find GMMA to be superior in defining the self-association model of oligomerization.

The use of multiple complementary techniques is very attractive and has proven highly valuable in many laboratories^2,11–13,15^. To facilitate leveraging complementary techniques through GMMA we have implemented analysis models for nonideal self-association in our shareware GMMA software SEDPHAT.

## Results

### Separate analyses of DLS, SLS, and SV

**Figure 1:**
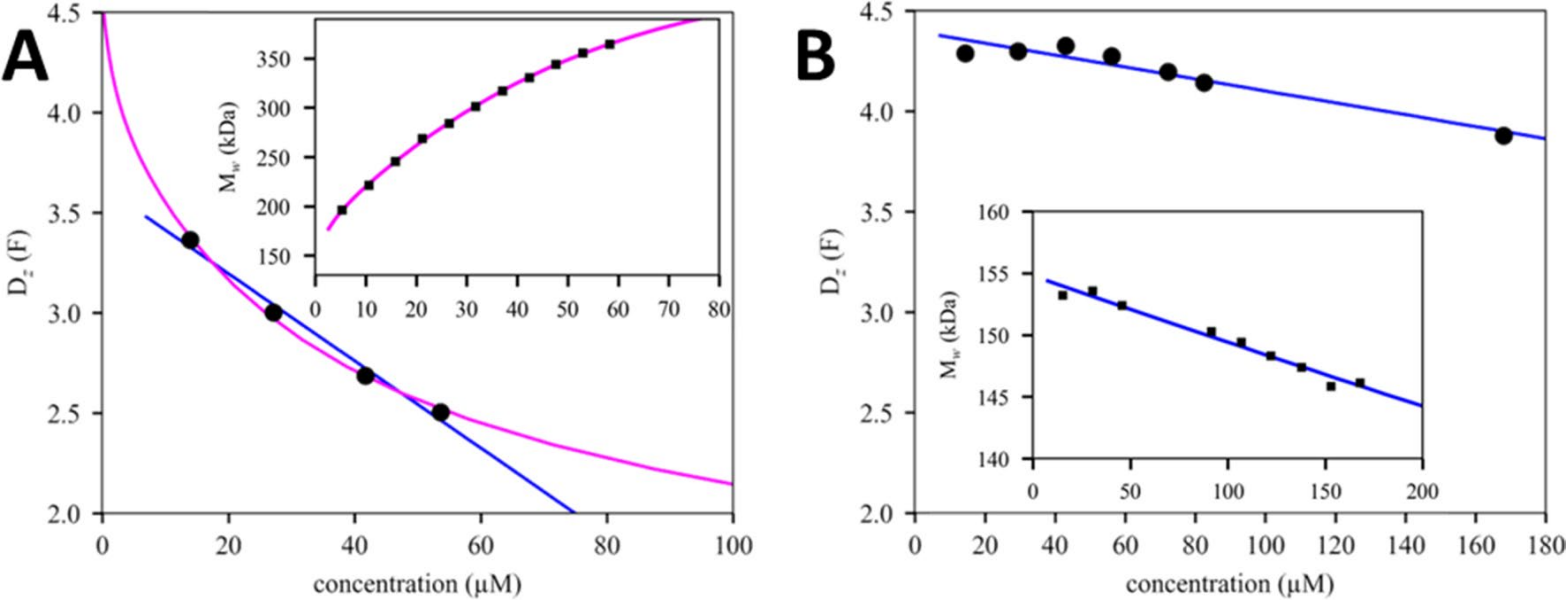
Single isotherms may lack range and resolution to discriminate self-association from nonideality. (**A**) z-average diffusion coefficients of the most strongly self-associating molecule (mAb C) measured by DLS (circles), with best-fit linear regression in blue, resulting in *k*_*D,op*_ =  −40.1 mL/g. Shown in magenta is the best-fit self-association model when jointly fitting the *M*_*w*_ isotherm from SLS (inset); this results in a best-fit *k*_*D*_ of − 6.6 mL/g. (**B**) z-average diffusion coefficients from DLS for mAb B that does not exhibit self-association (circles), with linear regression (blue line) jointly modeling the DLS data and the *M*_*w*_ isotherm from SLS (inset) as a single nonideal species with *k*_*D,op*_ =  −4.1 mL/g. Diffusion coefficients are reported in units of Ficks, with 1 F = 10^−11^ m^2^/s.

The dilemma of analyzing nonideal self-associating systems can be illustrated well considering the z-average diffusion coefficients measured in DLS. Figure 1A shows the DLS data from the most strongly self-associating molecule of our panel, mAb C. Linear regression (blue line) provides an operational nonideality coefficient of diffusion of − 40.1 mL/g. For the same sample, SLS shows a strong increase of weight-average molecular weights with concentration (inset), unequivocally demonstrating the presence of self-association. After self-association is accounted for, in a two-step isodesmic association model globally fit to DLS and SLS jointly (magenta line), a very different nonideality coefficient of diffusion of − 6.6 mL/g is obtained. From the similarity of the goodness of fit of the DLS data, the latter technique alone would not have allowed to distinguish these two interpretations. Even if self-association were considered, DLS data by itself would be unable to provide meaningful self-association parameters due to the limited information content of the data. Therefore analyses of DLS isotherms are conventionally restricted to operational interpretation.

This behavior of mAb C is contrasted by mAb B, which does not self-associate. Here we obtain an unambiguous description of nonideality (Fig. 1B). SLS shows decreasing apparent molecular weights with concentration (inset), and a both DLS and SLS data sets can be described well with linear concentration-dependence up to the highest measured concentrations of 40 mg/mL and 30 mg/mL for DLS and SLS, respectively (blue lines).

These examples illustrate the importance of the thermodynamic reference frame on the value of nonideality parameters in the presence of self-association, and how experimental resolution and concentration range may dictate the choice of the reference frame. Nevertheless, theoretical considerations outlined in the Methods show that either framework should provide self-consistent values of nonideality coefficients that obey 2*B*_2,op_ = *k*_S,op_ + *k*_D,op_, or 2*B*_2_ = *k*_S_ + *k*_D_, respectively.

To test this theory, Table 1 shows results from linear regression of the isotherms of DLS, SLS, and SV for the entire panel of mAbs (Table 1). One problem in the determination of operational parameters arises for mAbs that exhibit self-association, in that the *M*_*w*_ isotherms in SLS reveals self-association and requires fits with explicit self-association models. However, this problem can be remedied through the relationship *B*_2,op_ = *B*_2_ − *K* (Methods Eq. 14) that transforms virial coefficients back into the operational reference frame^27^. We find that, within experimental errors, 2*B*_2,op_ = *k*_S,op_ + *k*_D,op_ holds for mAb B, which does not self-associate. However, increasing discrepancies are obtained with greater extents of self-association: The operational virial coefficients from SLS deviate significantly from those predicted by combination of *k*_*S,op*_ and *k*_*D,op*_ from SV and DLS. Conversely, the operational nonideality of sedimentation is not predicted by apparent *B*_2_ and *k*_*D,op*_ from light scattering (Table 1). Thus, the validity of 2*B*_2*,op*_ = *k*_*S,op*_ + *k*_*D,op*_ cannot be confirmed experimentally, with discrepancies that can be so large as to render the operational parameters quantitatively meaningless. As illustrated in Fig. 1A for the most strongly self-associating mAb C, the errors may not be apparent in linear regression.

**Table 1:** Phenomenological non-ideality parameters from single method analyses and combinations.

mAb	k_S,op-SV_^(a)^ (mL/g)	k_D,op-DLS_^(b)^ (mL/g)	B_2,SLS_^(c)^ (mL/g)	SLS model	B_2,opSLS_ = B_2,SLS_ − K*^(d)^ (mL/g)	B_2,op-SV/DLS_^(e)^ (mL/g)	k_S,opLS_^(f)^ (mL/g)
B	6.9 (0.2)	− 4.5 (0.4)	1.13 (0.06)	1	1.13	1.2	6.8
A	4.3 (0.1)	− 9.6 (0.3)	− 1.70 (0.01)	1–2	− 3.9	− 2.7	6.2
E	− 3.8 (0.1)	− 16.1 (0.8)	− 7.7 (0.27)	1–2	− 18.8	− 10.0	0.7
D	− 0.6 (0.7)	− 7.0 (0.3)	− 0.36 (0.06)	1–2 and 2–4–…iso	− 25.8	− 3.8	6.3
C	− 38.4 (5.5)	− 40.1 (1.9)	5.5 (1.6)	1–2 and 2–4–…iso	− 216	− 39.3	51.1

^(a)^Operational nonideality coefficient from linear regression of *s*_*w*_(*c*) isotherms from SV without accounting for self-association. Values in parentheses are confidence intervals estimated from Monte-Carlo analysis.

^(b)^Operational coefficient from linear regression of z-average diffusion coefficients *D*_*z*_(*c*) observed in DLS.

^(c)^The second virial coefficient derived from fitting the *M*_*w*_(*c*) isotherm from SLS with a nonideal self-association model, in non-linear regression determining both best-fit binding constants *K* and *B*_2_.

^(d)^Operational second virial coefficient calculated with correction for self-association as in Eq. (17); using K* from best-fit nonideal binding model (for two-step models K* is calculated from the average of both binding constants).

^(e)^Predicted operational virial coefficient from combination of DLS and SV results as in Eq. (11).

^(f)^Predicted operational *k*_*S*_-value from combination of SLS and DLS as in Eq. (11).

**Figure 2:**
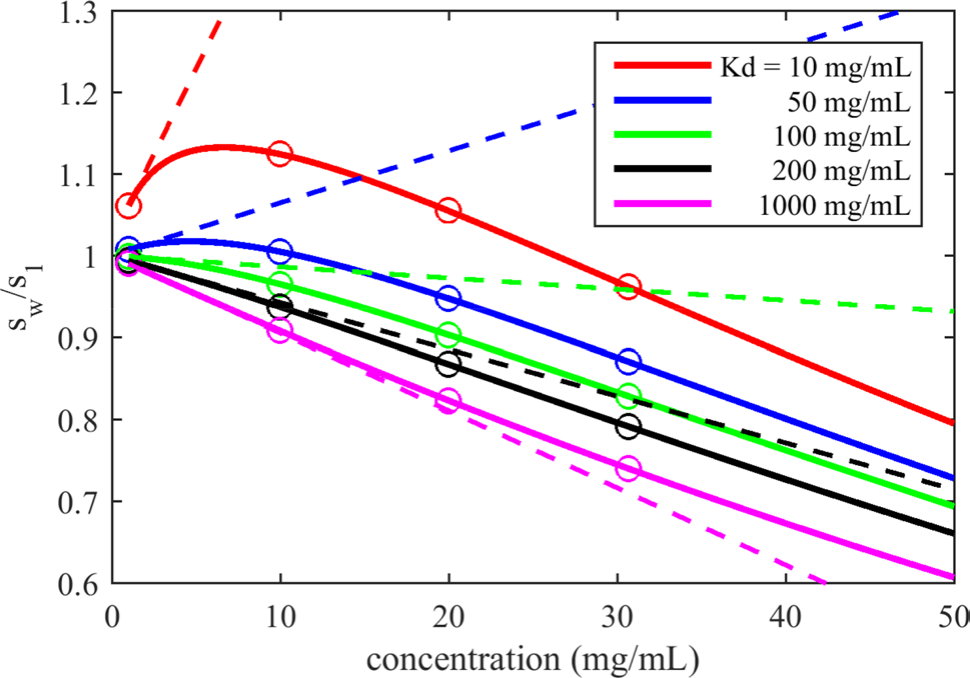
Isotherms sampled at experimental concentrations will not represent limiting slopes. Shown are isotherms of weight-average sedimentation coefficient of a protein in monomer–dimer self-association equilibrium with different equilibrium constants (color code in legend) with nonideality as predicted by the modified Richardson–Zaki expression by Fiore et al.^20^ assuming moderate shape asymmetry (solid lines). Circles represent potential measurements at concentrations spaced across experimentally accessible range. Slopes in the limit of low concentrations are depicted as dashed lines.

To explain the origin of the apparent discrepancy between theory and experiment, Fig. 2 shows a theoretical isotherm of weight-average sedimentation coefficients for a nonideal monomer–dimer self-association with different dimerization constants (indicated by different colors). The dashed lines represent the limiting slopes at vanishing concentrations, which are the theoretical basis of operational coefficients. With the goal of characterizing formulations with concentrations on the order of 100 mg/mL in mind, sound experimental reasoning dictates stretching the experimental concentrations (circles) over a range as large as possible to avoid amplification of statistical experimental errors. As a consequence, the measured slopes may be very different from the theoretical limiting slopes, and depend on particular experimental concentration choices. Within experimental errors, residual curvature in sampled isotherm values may not be recognizable for any but the strongest self-association. (Even when curvature is present, in the published literature it is usually chosen not to be scrutinized with the aim to adhere to the operational definition of the parameters.) Similar pictures would emerge from isotherms of diffusion coefficients or apparent molecular weights, as may be discerned from considering the limiting slope in Fig. 1A.

Thus, despite the theoretically appealing invariance of the often used relationship *B*_2_ = (*k*_*S*_ + *k*_*D*_)/2 for reference frames either purely phenomenological or explicitly accounting for self-association, in practice the experimentally obtained operational parameter values are highly skewed due to the need to measure at finite concentrations, rendering them of uncertain quantitative value. On the other hand, dependent on the technique, as illustrated with DLS data in Fig. 1, resolution and information content of isotherms may not allow determination of anything but operational parameters.

### Global multi-method analyses of DLS, SLS, and SV isotherms

The goal of GMMA is to take advantage of orthogonal observations in different techniques while bridging the differences in resolution and concentration ranges. Through global modeling, a consistent reference frame can be applied that incorporates both nonideality and self-association explicitly. SEDPHAT is a software tool that seamlessly allows fitting data from different techniques side-by-side in a programming-free graphical user interface. As previously described, simply by loading different data types, the necessary parameters specific to particular techniques are added, joining those parameters that describe molecular properties as defined in global binding models^25,26,28^. For the present work, we have extended SEDPHAT to incorporate consistent nonideality parameters, as well as a graphical user-interface to create new binding models by identifying reaction steps with or without isodesmic or isoenthalpic indefinite assemblies.

The experimental basis is the same set of SLS, DLS, and SV experiments examined above. With regard to SV, to maximize the information content we take as the starting point the isotherm of weight-average sedimentation coefficients *s*_*w*,0_ extracted from the recently introduced method for nonideal sedimentation coefficient distributions, *c*_*NI*_(*s*_0_)^29^. This method fits raw sedimentation boundaries and allows determining an average nonideality coefficient of sedimentation *k*_*S*_ separate from the sedimentation coefficient distribution by evaluating boundary anomalies associated with hydrodynamic nonideality. Simultaneously, the sedimentation coefficient distribution allows the quantitation of irreversible aggregates, and provides information on the size range of reversible oligomers^29,30^. Integration of *c*_*NI*_(*s*_0_) yields weight-average sedimentation coefficients *s*_*w*,0_ of sedimenting species corrected for hydrodynamic nonideality. We have recently demonstrated application of this method for mAbs of the current panel up to 45 mg/mL, and the end-point of the previous study, i.e., the resulting s_w,0_ isotherm and the *k*_*S*_-values, will serve as starting point here^24^.

We build on this SV data, interpreting previously determined *s*_*w*,0_ isotherms and *k*_*S*_ -values within the context of GMMA with *D*_*z*_-isotherms from DLS and *M*_*w*_-isotherms from SLS. To enforce a self-consistent model, *B*_2_ is the only adjustable nonideality parameter, with *k*_*S*_ predetermined and *k*_*D*_ implicitly calculated as 2*B*_2_ − *k*_*S*_. These parameters are combined with explicit self-association models treating equilibrium binding constants as additional unknowns. Further constraining the model is prior knowledge on the known molar mass from mass spectrometry. Finally, the monomer sedimentation coefficient is constrained to that measured in SV in dilute solution, with the exception of mAb C which exhibits strong self-association.

The mAb in our panel resembling most closely a ‘non-interacting’ molecule is mAb B. This data set can serve as a test for GMMA in the simplest case. In principle, for data points close to dilute conditions the solution behavior is independent of nonideality, and global analysis of SLS, DLS, and SV at low concentrations should be consistent with the known molar mass as predicted in the Svedberg equation (Eq. 13). However, a naïve global fit results in a poor description of the data (Supplementary Figure S1). The addition of a term for a trace of large aggregate substantially improves the fit: Assuming ad hoc a 10 MDa species as representative for large particles, at the best-fit concentration of only 0.07% (% of total weight concentration) reduces χ^2^_r,app_ of the global fit ≈3.4-fold. Similar improvement is found after inclusion of a term for irreversible dimer, at a fixed population of 5.7% as measured by SV in dilute solution. (Different values for mass and shape of the aggregate produce different best-fit % population, with otherwise largely identical results, indicating there is no detailed information on the aggregate in the data.)

**Figure 3:**
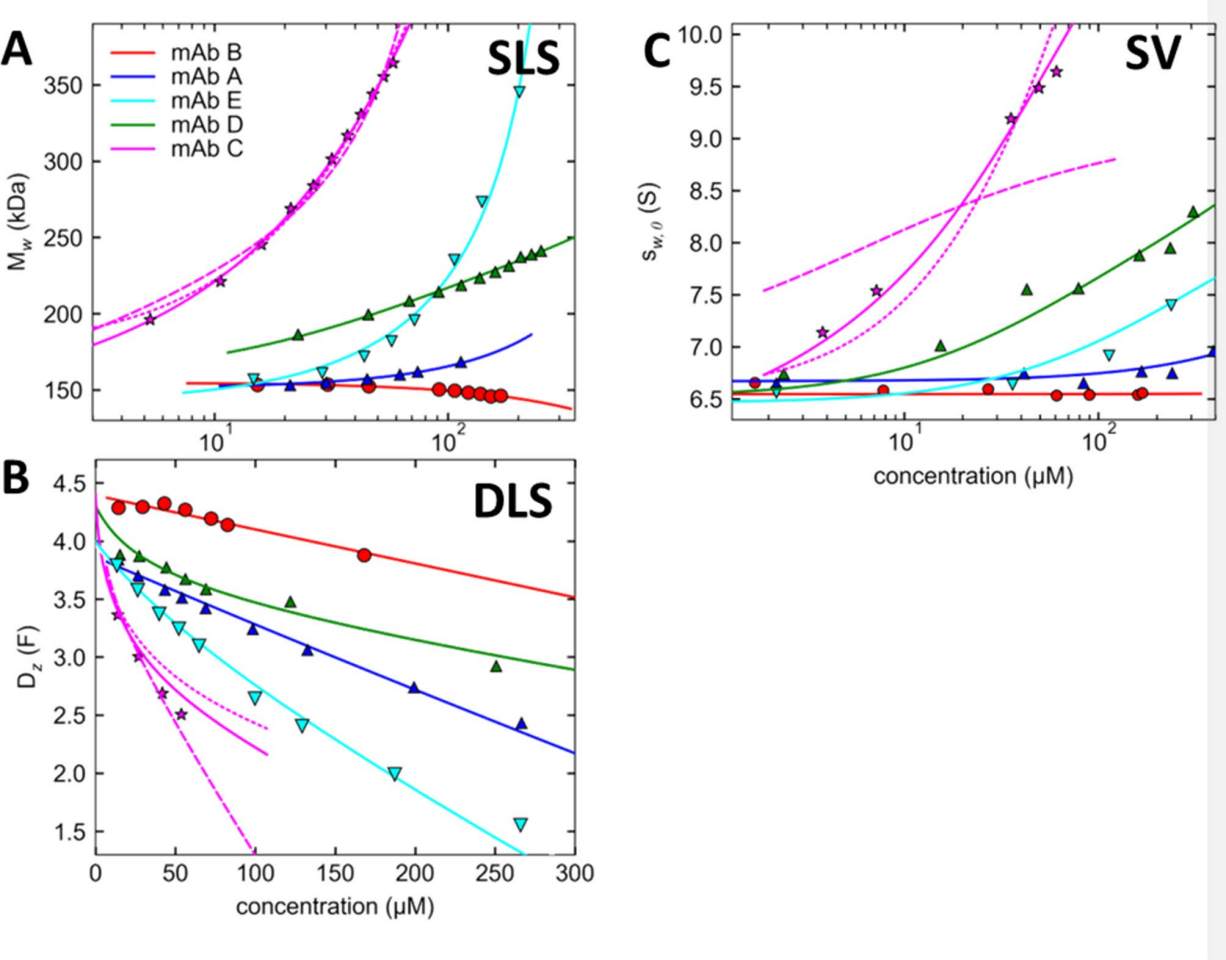
Global multi-method analysis with nonideal self-association binding models for the panel of mAbs. Simultaneous fit of SLS (**A**), DLS (**B**), and SV isotherm (**C**) with a model describing a nonideal monomer for mAb B (red), monomer–dimer self-association for mAb A (blue) and mAb E (cyan), monomer–dimer-tetramer-isoenthalpic self-association for mAb D (green), and monomer–dimer–tetramer self-association of mAb C (magenta, solid line). For comparison, best GMMA fit of mAb C data to a monomer–dimer model (dashed magenta line) and an isodesmic self-association model (dotted magenta line) are additionally shown. Best-fit parameter estimates underlying the models are in Table 2.

Finally, we allow for small errors in scattering contrast. This model provides an excellent global fit (Fig. 3) with approximately 20-fold reduced χ^2^_r,app_ at the best-fit scattering contrast correction of 4%. We believe this correction reflects unavoidable imperfections in our knowledge chiefly of the protein refractive index increment, and its contributions from amino acids, which differ from that of the carbohydrate component of the mAbs, and from the solvation shell^31,32^. Similar small uncertainties occur in the partial-specific volume, which we fix at 0.73 mL/g to avoid parameter correlations among the scattering and density contrast values. These systematic errors would be largely irrelevant and go unnoticed in single method fits, but become apparent when combining data in GMMA. Since at low concentrations these values are independent of any self-association and non-ideality, we consider this adjustment an experimental calibration factor for the GMMA. We apply the same, fixed correction values to all mAbs in the current study.

The isotherms and best-fit nonideal self-association models for all mAbs in GMMA are shown in Fig. 3, with results listed in Table 2. In most cases the fits are excellent considering they describe a set of orthogonal observables differing in resolution and information content, and jointly over-determine the nonideality parameters. The panel of mAbs covers different degrees of self-association versus nonideality, which offers further methodological insights.

**Table 2:** Best-fit binding parameters from GMMA of static and dynamic light scattering and sedimentation velocity.

mAb	Model	s_1_^(a)^ (S)	k_S,SV_^(b)^ (mL/g)	*K*_D,glob_	B_2,glob_ (mL/g)	k_D,glob_ (mL/g)	Irrev. dimer^(c)^ (wt%)	Irrev. aggreg.^(^^d)^ (wt%)
B	1	6.73	6.5 (0.2)	∞ (> 17 mM)	1.13 (0.12)	− 4.24	5.7	0.06 (< 0.01)
A	1–2	6.67	5.0 (0.3)	7.1 mM (5.9–8.5)	− 2.13 (0.11)	− 9.26	1.8	0.07 (< 0.01)
E	1–2	6.70	0.2 (< 0.01)	550 µM (510–730)	− 7.7 (0.2)	− 15.6	0.1	0.01 (< 0.01)
D	1–2 and 2–4…isoH	6.72	3.5 (0.2)	153 µM (136–169) and 5.3 mM (2.7– 75)	0.01 (0.23)	− 3.5	6.4	0.02 (0.03)
C	1–2 and 2–4	6.41 (0.16)	3.6 (2.2)	15 µM (14.7–21) and 48 µM (40–51)	− 5.9 (0.68)	− 15.4	0.4	0.0 (< 0.01)

Values in parentheses are confidence intervals or +/− errors, respectively, on a confidence level of 68.3%.

^(a)^The monomer *s*-value was determined from the *c*(*s*) analysis of sedimentation under ideal conditions at 0.3 mg/mL and fixed in the analysis, except for mAb C.

^(b)^Nonideal *c*_*NI*_(*s*_0_) analysis was carried out to pre-determine *k*_*S*_^24^.

^(c)^Irreversible dimer with assigned f/f_0_ = 1.8, at pre-determined population from SV in dilute solution, except for mAb E where best-fit parameters are shown.

^(d)^As a representation of very large trace aggregates, weight-percent of non-interacting species of 10 MDa with f/f_0_ = 1.8.

As mentioned above, mAb B (red) can be modeled well without self-association. Indeed, statistical analysis yields a lower limit for a monomer–dimer *K*_*D*_ of 17 mM. Nevertheless, negative *k*_*D*_ and the low *B*_2_-value clearly indicate soft attractive interactions (see Discussion). Interestingly, higher nonideality parameters cannot compensate for lower *K*_*D*_-values. This suggests that nonideality and self-association can be distinguished in GMMA.

Examining the parameter error estimates, it must be kept in mind that it is difficult to obtain absolute parameter errors in GMMA due to the dominance of systematic errors, although their relative values can be compared^25^. It can be discerned from Table 2 that the largest uncertainties in nonideality parameters appear for mAb C in the presence of the strongest self-association, where (in the absence of saturation of binding) nonideality is maximally masked. However, selection of the binding model is the most important factor contributing to uncertainties in *B*_2_. For example, for mAb C, GMMA arrives at the best fit for a monomer–dimer-tetramer model, slightly worse for monomer–dimer-trimer (with 25% higher *χ*^2^), and isoenthalpic self-association (with 75% increased *χ*^2^) (Table 3). For these models the best-fit *B*_2_-values scatter widely, with values of − 6.0 mL/g, − 9.5 mL/g, and − 5.0 mL/g, respectively. Models with more limited self-association, as well as those with more extended self-association, can be clearly excluded, as illustrated in dashed and dotted magenta lines in Fig. 3 for monomer–dimer and isodesmic self-association models, respectively. These data highlight the advantage of GMMA for discriminating among binding models: For example, a monomer–dimer model (dashed line in Fig. 3) provides a moderate fit to DLS and SLS, but can clearly be excluded based on a gross misfit of the SV data. While our main focus is on the nonideality parameters, it is evident that self-association parameters will be better defined in GMMA, not in the least from the higher confidence in the binding model.

**Table 3:** Discrimination of binding models from GMMA in comparison with analyses of data from single techniques.

mAb	Model	SV + SLS + DLS χ^2^_app_/χ^2^_best_	SLS χ^2^_app_/χ^2^_best_	SV χ^2^_app_/χ^2^_best_	SLS + DLS χ^2^_app_/χ^2^_best_
C	1–2	1.76	57	> 1000	52
	1–2–3	1.25	1.09	**1**	**1**
	1–2–4	**1**	**1**	101	3.3
	1–2–3–… isoenthalpic	1.74	1.56	360	3.8
	1–2–3–… isodesmic	4.5	1.09	549	7.9
D	1–2	1.08	1.36	**1**	1.3
	1–2–3–… isoenthalpic	2.97	3.17	2.4	3.8
	1–2–3–… isodesmic	3.38	3.05	2.5	4.7
	1–2–3	1.12	1.33	**1**	1.03
	1–2–4	1.07	1.18	**1**	**1**
	1–2 and 2–4–6–… isoenthalpic	**1**	**1**	**1**	1.12
E	1–2	1.07	**1**	3.3	**1**
	1–2–3 … isoenthalpic	**1**	1.57	**1**	1.47
A	1–2	**1**	**1**	**1**	**1**
	1–2–3 … isoenthalpic	1.02	830	1.17	1.38

Values shown are the factor increase of χ^2^_app_ relative to the χ^2^_app_ of the best-fit model marked in bold.

Interestingly, when examining the *χ*^2^-values for different models applied to single techniques, the statistical results show larger contrasts of different models (Table 3). This appears to be a result of the fewer data points involved and the outsized impact of single adventitious data points. However, monomer–dimer–tetramer and monomer–dimer–trimer models are consistently the two best for mAb C.

These results are mirrored for mAb D (green), where acceptable binding models are those with a moderate first dimerization step (*K*_*D*_ ≈ 150 µM), and these are improved by addition of significantly weaker higher-order association. It is noteworthy that the nonideality of mAb D in PBS is very close to the thermodynamic ‘theta-condition’ where the second virial coefficient vanishes^33,34^. This data offers opportunities for more detailed interpretation of the hydrodynamic nonideality (see Discussion). The best-fit self-association model features dimers that weakly, indefinitely self-assemble (Table 3). Across similarly well performing models, *B*_2_ estimates vary only by ≈0.3 ml/g, which is just slightly larger than the statistical error estimate in the best-fit (Table 2). The small variation reinforces the observation that stronger self-association obscures precision in nonideality parameters, and conversely, weaker self-association provides higher precision of assessing nonideality. Again, analysis of single techniques are consistent in the best binding models with GMMA. However, analysis of SLS alone, for example, yields best-fit *B*_2_*-*values differing by 1.4 mL/g among the two best models, which is a greater uncertainty than when analyzing the same data in the context of GMMA.

For mAb E (cyan) and mAb A (blue), still weaker self-association makes it more difficult to discriminate among different binding steps, and isodesmic or monomer–dimer models perform similarly well, with associated differences in best-fit *B*_2_ of 0.8 mL/g (mAb E) and 0.01 mL/g (mAb A), respectively. The decision between these models will depend slightly on the relative weight given to the different techniques. As observed with mAb C, single technique analyses suggest overly sharp contrast between binding models, which does not hold in the context of GMMA (Table 3). For example, modeling SLS data alone from mAb A with either monomer–dimer or isodesmic models yield 1000-fold differences in root-mean-square deviation (rmsd), yet either rmsd value is far below expected experimental errors.

In summary, these results demonstrate the advantage of GMMA for model selection, separation of self-association and nonideality, and parameter precision. Considering that SV is more time-consuming and perhaps a technically more challenging technique, the question arises to what extent GMMA of light scattering results alone—excluding SV data—can already garner the same advantages. An important advantage of GMMA as compared to separate DLS and SLS analyses is already demonstrated in Fig. 1A, which is the chance to impart a nonideal self-association model to the DLS analysis. This avoids gross errors apparent in operational nonideality parameters in Table 1. Best-fit parameters and error estimates from such limited GMMA are shown in Table 4, and comparison of the performance of different models is added in Table 3. Overall, similar results as in full GMMA are obtained, although usually exhibiting slightly larger parameter errors. Unfortunately, however, the limited GMMA does not perform reliably well for hydrodynamic nonideality parameters, as is apparent, for example, when comparing the implicit *k*_*S*_-values from the limited GMMA for mAb A and mAb C with the experimentally determined values in SV. While limited GMMA presents higher contrast in quality of fit among different self-association models, full GMMA highlights the greater susceptibility of a more limited set of techniques to systematic errors, and unequivocally assigns the best model based on all available data.

**Table 4:** Best-fit binding parameters from the GMMA of static and dynamic light scattering only.

mAb	Model	*K*_D,glob_	B_2_ (mL/g)	k_D_ (mL/g)	k_S,app_^(a)^ (mL/g)
B	1 + irrev. 2^(b)^	∞ (> 1.3 mM)	1.09 (0.06)	− 4.1 (0.4)	6.3 (0.41)
A	1–2	470 µM (420–530)	2.1 (0.5)	− 6.9 (1.0)	11.0 (1.4)
E	1–2… isoH	600 µM (530–660)	− 6.3 (0.4)	− 11.9 (0.7)	0.7 (1.1)
D	1–2 and 2–4–…iso	82 µM (76–86.3) and 9.0 mM (1.3–∞)	− 0.0 (0.73)	− 3.9 0.87)	3.9 (1.7)
C	1–2 and 2–4	16.0 µM (15.3–16.8) and 40.2 µM (36.8–44.1)	− 4.7 (0.6)	− 21.9 (0.8)	12.5 (1.4)

Values in parentheses are confidence intervals estimated from Monte-Carlo analysis.

^(a)^Apparent k_S_ calculated as k_S_ = 2B_2_ − k_D_.

^(b)^Irreversible dimer with best-fit population of 9% was required for the best fit.

## Discussion

It is widely recognized that the use of multiple techniques is highly useful for the characterization of protein self-association and nonideality^2,11–13^. However, reconciling the quantitative results from different techniques is generally complicated by different resolution and sensitivity. This is illustrated in the analysis of weak reversible self-association processes^14,35,36^. Dependent on solution concentration and technique used, transiently formed oligomers may be clearly discerned as populations of distinct species, or may only be indirectly apparent as a contribution to a nonideality coefficient such as the second virial coefficient *B*_2_.

This naturally leads to different choices how virial coefficients are defined, a problem that has been laid out long ago by Hill and Chen^27,37^. For a nonideal monomer–dimer system, for example, the second virial coefficient *B*_2_ may be defined after separately accounting for monomer and dimer populations in chemical equilibrium with equilibrium constant *K* , or it may be operationally defined to include all attractive interactions including those producing dimers, resulting in an apparent virial coefficient *B*_2_* = *B*_2_ – *K*^27,37^. As pointed out by Hill and Chen, the decision of which viewpoint to take cannot be made on the basis of thermodynamics, and both are valid. They are a priori indistinguishable, as long as linear terms in the concentration dependence are sufficient to describe experimental data. However, clarity on the framework is essential for comparing and interpreting parameter values.

In the present work we have extended these considerations to the nonideality coefficients of sedimentation and diffusion. These, too, may be either operationally defined including all concentration dependence (as seems unavoidable in DLS), or after separately accounting for self-association (which is usually possible at least to some degree in SLS and SV). A time-honored approximation is that nonideality is equal for all oligomers, and we consider obtained parameters an average of the different macromolecular assembly states^38^. Even though the parameter values in these frameworks will be very different, we have shown that in theory the operational coefficients obey their usual relationship with the equally operational second virial coefficient.

While this is, at first, a satisfying theoretical result, unfortunately we find it is practically not always useful since it holds only for limiting slopes that can be very different from slopes in linear regression of experimental data. In the extreme example of mAb C: the linear regression shown in Fig. 1A yields a *k*_D_ estimate of − 40.1 mL/g; for the same antibody a previously published^12^ linear regression covered half the concentration range yielding a value of − 59.6 mL/g; the true limiting value of the *D*_z_ isotherm would be − 424 mL/g; but after accounting for self-association separately in GMMA the best estimate of *k*_D_ is − 15.4 mL/g. (Published results for the different parameters and antibodies are listed in the Supplementary Table S1). This variability of values makes it very obvious that operational nonideality parameters in the presence of significant self-association may be quantitatively meaningless. Smaller discrepancies in operational *k*_D_ values are found for mAbs showing self-association only with *K*_D_ on the order of mM or higher. Thus, high-throughput determination of an operational *k*_D_ by DLS^12,39,40^ may still be very useful for qualitative screening purpose for the presence of self-association, but alone does not lend itself to quantitative interpretations. This problem will be exacerbated when comparing operational values from different techniques.

To enable reliable quantitation we can build on the fundamental classical observation that measurements of mass and transport in diffusion and sedimentation are orthogonal, and intimately linked. The use of the Svedberg equation to demonstrate accuracy of molecular parameters has a long history. In the present work we have extended this strategy to the global analysis of self-associating systems including nonideality, making use of both the Svedberg relationship and that between first-order nonideality coefficients 2*B*_2_ = *k*_S_ + *k*_D_. Crucially, the joint modeling of all techniques allows us to bridge the gap in resolution, and apply a consistent self-association model to all data.

We have previously introduced GMMA, and developed software for its convenient application, so as to take advantage of synergy between different techniques in the context of multi-site multi-protein interactions^25,26,28^. In the present application different techniques likewise provide synergistic advantages and limitations: SV has the highest resolution, and in the form of the recently introduced nonideal sedimentation coefficient distribution *c*_NI_(*s*_0_) separates oligomerization from nonideal sedimentation *k*_S_, the latter extracted from characteristic boundary anomalies^29,30^. We have shown recently how it can be applied to mAbs up to 45 mg/mL, which provides high sensitivity for self-association^24^. While SV gives clues to the self-association scheme and the polydispersity of the sample, a drawback is that it does not directly measure the molecular weights of oligomers, and has poorer information content on diffusion and *k*_D_, and therefore *B*_2_^29^. This is highly complementary to DLS, which contributes information on diffusion constants and *k*_D_, although it lacks oligomeric resolution and is most susceptible to trace aggregates. SLS measures molecular weights more directly, reports directly on *B*_2_, but has no information on hydrodynamic properties and only reports average values. All methods have in common the measurement of molecular states free in solution without significant dilution and without labels, and therefore must strictly adhere to the same model and jointly over-determine the set of nonideality parameters. Our GMMA results modeling data from the panel of antibodies show that this is indeed the case, that the assumptions underlying the models are consistent with the data, and that the GMMA analysis fully captures the joint information content.

An expected limitation in GMMA is the difficulty to obtain estimates for parameter uncertainties^25^. GMMA does rely on much larger number of data points than single techniques, however, it also exposes residual systematic errors between different techniques that hinder rigorous statistical analysis^25^. Unavoidable systematic differences, for example, in sample equilibration time, concentration measurements, and susceptibility of experiments to various sample imperfections or technical measurement imperfections invariably cause GMMA fits to each data set to be of lower quality than each individual fit. One could regard this as a useful ‘reality check’ that prevents over-interpretation of limited analyses. Another problem related to systematic errors is the estimated uncertainties assigned to each experiment that control their relative weight in GMMA. As discussed previously^25^, it is useful to verify that the results are not sensitive to these weights. On the other hand, for our examples of moderate or strong self-association, we found the largest source of uncertainty arises from model selection. Even though GMMA clearly benefits model selection, the latter still presents an important limitation. This may be addressed with a priori structural information, and/or extension of GMMA to include data from additional techniques and at higher concentrations. However, the present GMMA approach describes only first-order coefficients of nonideality, and therefore in the current form is bound to fail in the application to more highly concentrated solutions^20,33,41,42^.

With regard to the absolute values of the nonideality parameters we observed, it is striking that after accounting for all apparent self-association processes, the nonideality coefficients of diffusion are still always negative, and second virial coefficients are significantly below the value for hard sphere repulsion. It is interesting to note that these soft attractive components cannot be captured well in GMMA in the form of ultra-weak self-association, despite the expected ambiguity in reference frame at least far below any saturation implicit in a self-association picture.

In the context of SLS analysis, models have been proposed that reduce nonideality to describing volume exclusion by an effective hard sphere^43,44^. In cases where *B*_2_ < 0, such as mAbs A, E, and C, the associated effective radii would need to assume negative values, which would seem to defeat the intuitive aspects of such an effective hard particle picture. However, a hard particle excluded volume can be extended with the picture of non-uniform macromolecular distance distributions, that in turn modulate mutual hydrodynamic interactions, and thereby explain the observed behavior in concordance with well-established statistical fluid mechanics theory^20–22,45,46^. This picture will benefit greatly from measuring both hydrodynamic and thermodynamic nonideality parameters.

**Figure 4:**
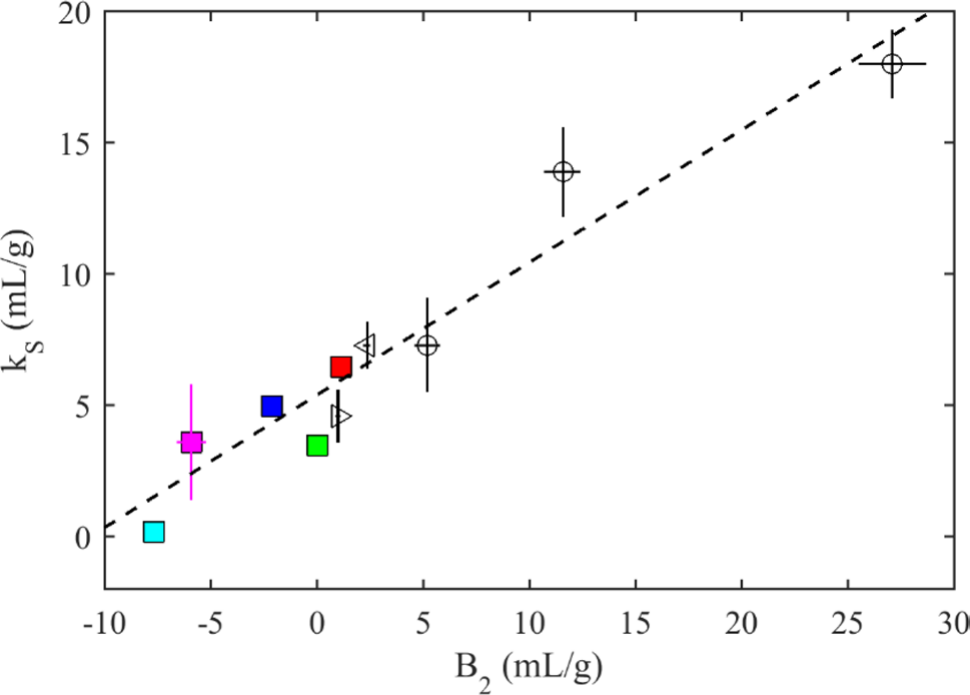
Relationship between hydrodynamic nonideality and second virial coefficients. Shown are the pairs of *k*_S_ and *B*_2_ obtained by GMMA for the panel of mAbs (squares, mAb B in red, mAb A in blue, mAb E in cyan, mAb D in green, and mAb C in magenta), and our previously measured values^33^ for two VRC IgGs (triangles) and NISTmAb in different solution conditions (circles). Statistical fluid mechanics theory predicts a relationship *k*_S_ = (3.03 + 3.52[*B*_2_/*B*_HS_])v*^20,21^. The joint linear regression of all IgG data (dashed line) leads to a *k*_*S*_ value at ‘theta’ conditions (where *B*_2_ = 0) of 5.4 mL/g and a slope of 0.505, implying v* = 1.8 mL/g and *B*_HS_ = 12.4 mL/g.

Even though hydrodynamic nonideality parameters *k*_*S*_ and *k*_*D*_ and the second virial coefficient *B*_2_ are represented by two separate fitting parameters in GMMA, statistical fluid mechanics predicts a direct relationship between *k*_*S*_ and *B*_2_, rooted in the fact that macromolecular distance dependence of hydrodynamic interactions is governed by the same interparticle potential as the second virial coefficient^20,21,47,48^. We and others have previously examined the relationship between experimental hydrodynamic nonideality parameters (*k*_*S*_ or *k*_*D*_) and the second virial coefficient of mAbs^33,39,49–51^. Figure 4 shows the present results from GMMA of the panel of mAbs in the context of our previous data from other IgG antibodies^33^. The expected linear relationship can be discerned, with a caveat from the fact that it assumes similarity of macromolecules whereas all IgGs are slightly different in mass, shape, and charge.

One particularly interesting case is mAb D, which at *B*_2_ ≈ 0 represents ‘theta’-conditions (Table 2). At this point *k*_*Dθ*_ and *k*_S*θ*_ are of equal magnitude but opposite sign, here ≈3.5 mL/g. Based on theory of hydrodynamic interactions on diffusion coefficients by Anderson and Reed^52^, which does not consider volume exclusion contributions^48^, it has been argued that under these conditions negative *k*_*D*_-values signify attractive interactions only if they are less than − 5.3 mL/g^34,53^. However, considering the fact that *B*_2_ is also less than the obligatory excluded volume repulsion, we would argue that compensating attractive interactions must obviously be at play. From the widely cited statistical fluid mechanics work of Batchelor, the threshold for *k*_*D*_ to indicate attractive interactions is at *k*_*D*_ <  + 1.45 × v* (with v* an effective hydrodynamic volume that for spherical particles would coincide with the partial-specific volume and otherwise be larger)^47,48^.

The measured coefficients under ‘theta’-condition allows us to determine the effective hydrodynamic volume. For the regression of Fig. 4 we find v* ≈ 1.8 mL/g, or ≈ 60% the volume of a sphere with the Stokes radius *R*_S_ and volume *V*_S_ (using the translational friction ratio *f*/*f*_0_ of ≈1.6 experimentally determined in SV in dilute solution). From the slope we can estimate the hard-sphere virial coefficient, which is 12.4 mL/g or 4% larger than 4*V*_*S*_. While we have taken the Stokes radius here solely as a convenient size standard to demonstrate that the measured values are reasonable, these experimental values may be compared with more refined structural models for antibodies and interpreted in the context of inter-particle potentials^45,54^. For example, knowing *B*_HS_ and v* the values of *B*_2_ may be interpreted in the Baxter adhesive sphere model, where a stickiness parameter 1/τ = (1 − *B*_2_/*B*_HS_)/4 measures the attractive interaction strength at contact^46,55^. Values in the range 6.7 < 1/τ < 16 have been associated with crystallization propensity^46^. For mAb B that does not significantly self-associate, we find 1/τ = 0.23. More detailed interpretation is out of the scope of the present work.

In conclusion, we believe that by being able to distinguish between reversible self-association producing distinct oligomeric complexes, and attractive or repulsive interactions modulating the molecular distance distribution, GMMA can provide more meaningful quantitative measurement of nonideality parameters. This removes an existing bottleneck and better supports molecular interpretation of nonideality coefficients, to understand and predict protein states in concentrated solutions.

## Methods

### Theoretical description of nonideal sedimentation and diffusion for self-associating systems

Nonideality coefficients of sedimentation *k*_*S*_ describe in first-order approximation the concentration-dependence of sedimentation coefficients

1 $$ s(w) = s_{0} (1 - k_{S} w) $$

with *s*_0_ denoting the sedimentation coefficient in infinite dilution, and *w* denoting the protein concentration in weight units of mg/mL. We adopt weight concentrations in the discussion of nonideality since this directly relates to the solution fraction occupied by macromolecules, $\Phi = \overline{v}w$, that scales nonideality effects. (We prefer molar concentrations *c*_*i*_ in the context of self-association, which is governed by the number density of molecules, with the interconversion *w*_*i*_ = *ic*_*i*_*M*_1_). We may consider the negative slope of *s*(*w*) at low concentrations as an operational definition of the nonideality coefficient

2 $$ k_{S,op} = - \mathop {\lim }\limits_{w \to 0} \left[ {\frac{1}{{s_{0} }}\frac{ds}{{dw}}} \right] $$

We can apply this to the case of an interacting system where concentration dependence does not only arise from hydrodynamic interactions but also due to reversible complex formation. For simplicity we assume a monomer–dimer system, where monomer at concentration *w*_1_ and dimer at concentration *w*_2_ follow mass action law

3 $$ w_{2} = 2K^{\prime}w_{1}^{2} $$

with *K’* denoting the molar equilibrium association constant *K* converted to weight units ($K^{\prime} = {K \mathord{\left/ {\vphantom {K M}} \right. \kern-\nulldelimiterspace} M}$ , with *K* defined in molar units with mass action law $K = {{c_{2} } \mathord{\left/ {\vphantom {{c_{2} } {c_{1}^{2} }}} \right. \kern-\nulldelimiterspace} {c_{1}^{2} }}$ between monomer and dimer molar concentrations *c*_1_ and *c*_2_). A more precise description of the monomer–dimer equilibrium would consider the concentration-dependence of chemical activity coefficients; however, in the limit *w* → 0 considered here these terms would vanish and are therefore omitted for clarity. Likewise, we could consider higher oligomerization processes, but in the limit of low concentration contributions from terms higher power in *w* than dimerization will disappear. Therefore, we can focus in the following derivation on the effect of reversible dimerization. In the absence of hydrodynamic nonideality one would observe a weight-average sedimentation coefficient of

4 $$ s_{w,0} = \frac{{s_{1} + s_{2} 2Kw_{1} }}{{1 + 2Kw_{1} }} $$

For molecules in fast exchange, this *s*_*w,*0_ corresponds to their time-average sedimentation velocity. Nonideality of sedimentation arises from the finite volume occupancy of sedimenting particles causing solvent backflow, and from the long-range nature and non-additivity of particle hydrodynamic flow fields. For randomly distributed spheres, statistical fluid mechanics predicts a fractional retardation of sedimentation by 6.55-fold the occupied volume fraction^17^ (which for proteins may be approximated as the volume of the hydrodynamically equivalent spheres^33,56^). With experimental precision of *s*-values in the order of 0.1%, for antibodies this hydrodynamic nonideality typically becomes significant at concentrations in excess of ≈1 mg/mL. For non-randomly distributed particles the fractional retardation is modulated by the factor (1 + 1.16*B*/*B*_HS_)^33^. In first-order approximation we adopt the time-honored simplification that different oligomers exhibit the same nonideality coefficient *k*_*S*_^38^. Thus, the measured sedimentation coefficient will be

5 $$ s_{w} = \frac{{s_{1} + 2s_{2} Kw_{1} }}{{1 + 2Kw_{1} }}\left( {1 - k_{S} \left( {w_{1} + 2Kw_{1}^{2} } \right)} \right). $$

The operationally defined *k*_*S,op*_-value (based on Eqs. 2 and 4, using the chain rule and taking the limit *w* → 0) will be

6 $$ k_{S,op} = k_{S} - 2\frac{{s_{2} - s_{1} }}{{s_{1} }}K $$

This is the phenomenological nonideality coefficient one would obtain from the slope in the plot of measured *s*-values as a function of total concentration in the limit of low concentration. It requires that all concentrations are below the equilibrium dissociation constant for dimerization. In that case, dimerization is far from saturation and little indication of nonlinearity is presented in the isotherm.

Analogous to the case of sedimentation, nonideality coefficients of diffusion *k*_*D*_ describe in first-order approximation the concentration-dependence of diffusion coefficients

7 $$ D(w) = D_{0} (1 + k_{D} w) $$

with *D*_0_ denoting the diffusion coefficient in infinite dilution. It is typically measured as the slope *D*(*w*) leading to an operational definition of the nonideality coefficient of diffusion

8 $$ k_{D,op} = \mathop {\lim }\limits_{w \to 0} \left[ {\frac{1}{{D_{0} }}\frac{dD}{{dw}}} \right] $$

For the same monomer–dimer system outlined above, by most techniques it would be impossible to resolve monomer and dimer. For example, by DLS the measured diffusion coefficient is the *z*-average

9 $$ D_{z} = \frac{{D_{1} + 4D_{2} Kw_{1} }}{{1 + 4Kw_{1} }}\left( {1 + k_{D} \left( {w_{1} + 2Kw_{1}^{2} } \right)} \right) $$

With the definition of Eq. 8, the operationally defined apparent nonideality coefficient of diffusion is

10 $$ k_{D,op} = k_{D} - 4\frac{{D_{1} - D_{2} }}{{D_{1} }}K $$

Similar to *k*_*S,op*_, since diffusion coefficients of oligomers decrease with size, *k*_*D,op*_ can assume values in the presence of self-association that are smaller than in the non-interacting case.

In the absence of self-association, virial expansion of the osmotic susceptibility leads to the well-known relationship between second virial coefficient and the nonideality coefficients of sedimentation and diffusion^16^

11 $$ B_{2} = {{\left( {k_{S} + k_{D} } \right)} \mathord{\left/ {\vphantom {{\left( {k_{S} + k_{D} } \right)} 2}} \right. \kern-\nulldelimiterspace} 2} $$

(It should be noted that some authors prefer a definition of *k*_*S*_ based on solution density corrected sedimentation coefficients, with introduces the partial-specific volume as an additional term in Eq. 11; in the present work we assume no solution density or viscosity correction is applied, which leads to the form Eq. 11^16,29,57^. Both definitions are equivalent if applied consistently, but the latter form is consistent directly with statistical fluid mechanics results and with results of the nonideal *c*(*s*) distribution^29^).

It is of interest to compare this virial coefficient value with what would be obtained using the apparent nonideality coefficients of sedimentation and diffusion

12 $$ B_{2,op}^{*} \equiv {{\left( {k_{S,op} + k_{D,op} } \right)} \mathord{\left/ {\vphantom {{\left( {k_{S,op} + k_{D,op} } \right)} 2}} \right. \kern-\nulldelimiterspace} 2} $$

Using the Svedberg equation

13 $$ \frac{s}{D} = \frac{{M\left( {1 - v\rho } \right)}}{RT} $$

and inserting Eqs. 6 and 10 into Eq. 12 leads to

14 $$ B_{2,op}^{*} = B_{2} - K $$

Previously, Hill and Chen have examined the impact of self-association on similarly operationally defined second virial coefficient, which may be measured directly, for example, by sedimentation equilibrium^27,37^. In this case, it was also found that the apparent virial coefficient decreases with stronger self-association,

15 $$ B_{2}^{*} = B_{2} - K $$

This result is consistent with the hydrodynamic measurements: It shows that whether (1) self-association is operationally considered part of the macromolecular distance distribution reflected in B*_2_ and B*_2*,op*_; or, alternatively, (2) self-association is accounted separately as distinct complexation event leaving *B* to describe exclusively steric repulsion and far-field electrostatic interactions—in both cases will the average between *k*_*S*_ and *k*_*D*_ equal the virial coefficient from thermodynamic measurements. However, this requires consistent definitions of nonideality coefficients and virial coefficients.

The previous result was derived only in the dilute limit. At finite concentrations the linear approximation of the concentration-dependent observables breaks down. However, this can be captured with serial expansion of the saturation curve described by mass action law, which in turn can be mapped on to higher-order virial expansions. In this way, ideal monomer-*N*-mer self-association may be described in a framework of virial coefficients as the infinite series^58^

16 $$ B_{j(N - 1) + 1} = \left( { - 1} \right)^{j} \frac{(jN - 1)!}{{j!\left[ {jN - (1 + j)} \right]!}}K^{j} $$

For example, and ideal monomer–dimer system, it is $B_{2} = - K$, $B_{3} = 3K^{2}$, $B_{4} = - 10K^{3}$, $B_{5} = 35K^{4}$, etc. However, the series converges only for small fractional saturation, below 25% for monomer–dimer systems and less for higher-order oligomerization^58^.

In the present context, a consequence is that the relationship between the phenomenological picture not explicitly accounting for self-association and the framework considering self-association, expressed in relationships Eqs. (6), (10), and (14) will break down at finite concentrations. For finite, low fractional saturations, description of the concentration-dependent behavior of self-association in the picture of virial expansion requires a series of higher-order virial coefficients.

### Global multi-method analysis of nonideal self-association

GMMA was carried out according to previously described principles in the multi-method analysis program SEDPHAT^25,26^. Briefly, it rests on a unique set parameters {p_glob_} describing molecular behavior, which foremost include cumulative binding constants *K*_*X*_ (or free energies of binding) for formation of *N* complexes at concentration *c*_x_, each composed of *n*_*m,x*_ copies of component *m*. The total number of components *M* for self-associations is 1, but in the current implementation of SEDPHAT can be up to 4. Given the total component concentrations for all experimental isotherm data points, mass action laws for all species and overall mass conservation are used to calculate all species populations:

17 $$ \begin{aligned} c_{m,tot} = c_{m,free} + \sum\limits_{x = 1}^{N} {n_{m,x} c_{x} } + c_{m,irr} \hfill \\ c_{x} = K_{x} \prod\limits_{m = 1}^{M} {c_{m,free}^{{n_{m,x} }} } \hfill \\ \end{aligned} $$

(with *c*_*m,irr*_ accounting for potential small fractions of component *m* that are not participating in reversible association, being either binding incompetent conformation or sequestered in irreversible aggregates.)

In order to achieve a flexible and convenient programming-free modeling platform, a model editor was created that allows different self- or hetero-association schemes to be specified either via sequential reaction steps or directly as cumulative equilibrium constants from free species of constituent components for complexes with specified composition. This includes indefinite self-association pathways. Their numerical execution is truncated to a user-determined term, which can be assessed based on the weight-percent of material represented in the highest-order term. In the current work this is kept < 0.1%, beyond which we did not observe differences in the quality of fit. Isoenthalpic self-association, described by Chatelier^59^, was implemented as a modulation of isodesmic self-association, where different extent of translational and rotational entropy of the system in each association step dependent on oligomer size are taken into account. This leads to a stronger decay in the abundance of sequentially higher-order oligomers^59^. In the current implementation, this relies on ellipsoidal models of shape based on sedimentation coefficient and molecular weight of oligomers. All indefinite association schemes are based on hydrodynamic scaling laws assuming user-defined power-coefficient, in the present work taken as 2/3 for globular particles.

Species concentrations for each isotherm data points are used to model different observables for each data set, incorporating technique-specific features as needed, which may include additional fitting parameters {p_loc_}. The objective function for global optimization is an apparent reduced chi-square, χ^2^_r,app_, which represents the weighted sums of squared residuals of all data sets

18 $$ \chi_{r,app}^{2} = \frac{1}{{\sum\nolimits_{e} {N_{e} } }}\sum\nolimits_{e} {\sum\nolimits_{i = 1}^{{N_{e} }} {\frac{{\left( {y_{e,i} - f_{e,i} (\{ p_{glob} \} ,\{ p_{loc,e} \} )} \right)^{2} }}{{\left( {w_{e} \sigma_{e,i} } \right)^{2} }}} } $$

(for experiments indexed *e* with *N*_*e*_ data points each from measurements *y*_*e,I*_ with statistical error *σ*_*e,i*_ and systematic error of the method *w*_*e*_, fit with a model *f* that depends on global parameters p_glob_ describing the molecular properties and potentially on local parameters p_loc,e_ related to the specific experiment)^25^.

SEDPHAT is organized so that data sets of different types can be added in any number and sequence, and appropriate models and (if necessary) secondary model-specific parameters are added automatically, allowing for flexibility of shared parameters such as molecular extinction coefficients or signal quenching parameters. SEDPHAT is used in global modeling of sedimentation equilibrium and velocity analytical ultracentrifugation, isothermal titration calorimetry, autocorrelation functions from DLS, steady-state surface plasmon resonance surface binding and surface competition, and signal isotherms from various types of optical spectroscopy. In the present work we have extended the software to accommodate isotherms of weight-average molecular weights from SLS, and isotherms of z-average diffusion coefficients from DLS. SEDPHAT version 16 can be downloaded freely at https://sedfitsedphat.nibib.nih.gov/software.

The measurement error *w*_e_σ_e,I_ was assessed based on estimated variance of replicate measurements, and taken as 0.4 kDa for SLS, 0.02 F for DLS, and 0.03 S for SV. As analyzed in detail previously^25^, due to the uncertainties in the standard deviations of data acquisition for the different data types and the unavoidable impact of systematic errors, in practice one cannot expect that the reduced chi-square normalizes to a value of 1.0 for a seemingly perfect fit, as would be the case ideally in theory. However, the empirical values for χ^2^_r,app_ can still be used to assess relative quality of fit for different models, such as in F-statistics.^25^ In the present work, unless otherwise mentioned, we use Monte-Carlo analysis with 1000 samples and determine the central 68.3% quantile equivalent to one standard deviation. F-statistics analysis and Monte-Carlo analysis lead to somewhat different magnitude error estimates, which is a result of actual adventitious errors in the experimental data points differing from expected random variation assumed in the Monte-Carlo statistics; this difference is exacerbated in data sets with few data points. For error propagation the variance formula was used.

Global parameters in the GMMA models in the present work include, besides the binding constants, irreversible aggregate fractions, the virial coefficient *B*_2_, the nonideality coefficient *k*_*S*_, and species’ molecular weight and *s*-values. All species’ molecular weights were calculated from complex composition, diffusion coefficients were determined from each species molecular weight and *s*-value via the Svedberg equation using partial-specific volume of 0.73 mL/g, and *k*_*D*_ was implicitly constrained as *k*_*D*_ = 2*B*_2_ − *k*_*S*_. Unless otherwise mentioned only the binding constants, virial coefficient, and trace aggregate fraction were adjusted in the global fit. Molecular weights are known, irreversible dimer fraction are from SV in dilute solution, as are the monomer *s*-value and an estimated s-value of 9.5 S for the dimer. Higher oligomer *s*-values were estimated on a 2/3-power scaling law. Isotherms were plotted with GUSSI^60^ (kindly provided by Dr. Chad Brautigam).

### Antibodies

mAbs A–E are provided by AstraZeneca after a series of purification and chromatography steps. All mAbs were of subclass IgG1 except mAb D (IgG2). Final purified protein was dialyzed into PBS (137 mM NaCl, 2.7 mM KCl, 10 mM Na_2_HPO_4_, and 1.8 mM KH_2_PO_4_, pH 7.4). Concentration was determined using composition-based predicted extinction coefficient at 280 nm, but finally measured for each sample in SLS and SV by refractometry.

### Dynamic light scattering

DLS was carried out using a 384-well plate DynaPro DLS instrument (Wyatt Technology, Santa Barbara, California) equipped with a 830-nm laser and digital autocorrelator. 35 μL of sample was loaded into each well and the plate was spun at 2000 rpm for 30 s to remove any air bubbles prior to measurement at 20 °C or 25 °C, respectively. The z-average translational diffusion coefficient was determined from cumulant analysis of the autocorrelation function^61^, and modeled as

19 $$ D_{z} (c_{tot} ) = \left( {1 + k_{D} M_{1} c_{tot} } \right)\frac{{\sum\nolimits_{i} {c_{i} M_{i}^{2} D_{i} } }}{{\sum\nolimits_{i} {c_{i} M_{i}^{2} } }} $$

using species’ molar concentrations as predicted from sample composition and mass action law (Eq. 17), with species’ diffusion coefficients *D*_*j*_ = *s*_*j*_RT/*M*_*j*_(1-vρ) and approximating nonideality with a single average nonideality coefficient and total protein weight concentration $w_{tot} = M_{1} c_{tot}$.

### Static light scattering

SLS experiments were performed with a Calypso automated dilution system connected in series to a Dawn Heleos II instrument (Wyatt technologies, Santa Barbara, CA), combined with a refractive index detector (Optilab Rex, Wyatt technologies, Santa Barbara, CA). Measurement of excess light scattering were carried out at 20 °C or 25 °C, and converted into apparent molecular weight units by normalization with concentration and with the optical constant *K* = 4π^2^*n*^2^(d*n*/d*c*)^2^/*N*_A_*λ*^4^ with *n* denoting solvent refractive index (1.33), *dn/dc* the refractive index increment (taken as 0.185 mL/g), *N*_A_ the Avogadro number, and *λ* the wavelength of the light (661 nm). This was modeled as

20 $$ M_{w,app} (c_{tot} ) = \frac{1}{{1 + 2B_{2} M_{1} c_{tot} }}\frac{{\sum\nolimits_{i} {c_{i} M_{i}^{2} } }}{{\sum\nolimits_{i} {c_{i} M_{i} } }} $$

using species’ molar concentrations as predicted from (Eq. 17).

### Sedimentation velocity

SV experiments were carried out using a ProteomeLab analytical ultracentrifuge (Beckman Coulter, Indianapolis) as described previously^24^. Briefly, samples in concentrations ranging from 0.2 to 46 mg/ml were diluted from stock in PBS and loaded into cell assemblies with sapphire windows and two-sector Epon or 3D printed short-pathlength centerpieces^62^. All cell assemblies were placed in an 8-hole An-50 Ti rotor, and temperature equilibrated at a set point of 20 °C for at least 2 h. Data acquisition commenced using the Rayleigh interference optical detection system after acceleration to 45,000 rpm. Collected scans were corrected for scan time errors^63^ using the software REDATE (kindly provided by Dr. Chad Brautigam), and fit to the *c*_NI_(*s*_0_) model^29^ in SEDFIT versions 16.2 to 16.3p4. *s-*values were corrected for temperature and radial calibration errors^31^.

The signal-weighted average sedimentation coefficients are generally described with a single nonideality coefficient *k*_S_, since it is not possible to resolve separate nonideality coefficients for different species. This leads to the isotherm

21 $$ s_{w} (c_{tot} ) = \left( {1 - k_{S} M_{1} c_{tot} } \right)\frac{{\sum\nolimits_{i} {c_{i} \varepsilon_{i} s_{i} } }}{{\sum\nolimits_{i} {c_{i} \varepsilon_{i} } }} $$

with species molar signal coefficients ε_i_ and sedimentation coefficients *s*_*i*_^57^. Since the *s*_w_-values in the current work are obtained from integration of nonideality-corrected sedimentation coefficient distributions *c*_NI_(*s*_0_)^29^ after fitting raw sedimentation boundary profiles, the nonideality term in Eq. 21 is not necessary anymore. With molar signal coefficients being proportional to the species molecular weight the isotherm model simplifies in the present context to

22 $$ s_{w,0} (c_{tot} ) = c_{tot}^{ - 1} \sum\limits_{i} {ic_{i} s_{i} } . $$

For GMMA, *k*_S_ is fixed to the best-fit value from the same previous *c*_NI_(*s*_0_) analysis. Error estimates for *k*_S_ are from the error projection method with F-statistics on a 68.3% confidence level.

## Supplementary Information

Supplementary Information.
