# Supplementary material for: Global multi-method analysis of interaction parameters for reversibly self-associating macromolecules at high concentrations
Source: Sci Rep. 2021 Mar 11;11:5741. doi: 10.1038/s41598-021-84946-8 (PMC7952917; doi:10.1038/s41598-021-84946-8)
Supplement: Supplementary file 1 — Supplementary Information. [file 41598_2021_84946_MOESM1_ESM.pdf]

Global multi-method analysis of interaction parameters for reversibly self-associating macromolecules at high concentrations

Arun Parupudi[a], Sumit K. Chaturvedi[b], Regina Adão[b], Robert W. Harkness[c], Sonia Dragulin-Otto[a], Lewis E. Kay[c,d], Reza Esfandiary[a], Huaying Zhao[b], Peter Schuck*[b]

[a]Department of Dosage Form Design and Development, Biopharmaceuticals R&D, AstraZeneca, Gaithersburg, Maryland 20878, USA. [b]Dynamics of Macromolecular Assembly Section, Laboratory of Cellular Imaging and Macromolecular Biophysics, National Institute of Biomedical Imaging and Bioengineering, National Institutes of Health, Bethesda, Maryland 20817, USA. [c]Departments of Molecular Genetics, Biochemistry, and Chemistry University of Toronto, Toronto ON M5S 1A8, Canada. [d]The Hospital for Sick Children Research Institute, Toronto, Ontario, M5G 0A4, Canada.

* for correspondence: peter.schuck@nih.gov

Supplementary Information

Supplementary Figure S1

Supplementary Table S1

Supplementary Figure S1

Global analysis of M_w-isotherms (Panel A), D_z-isotherms (Panel B), and $s_{w,0}$-isotherms (Panel C) of mAb B with a single nonideal species model. The dashed line shows the global best-fit based on mass spectrometric molar mass of 148.7 kDa, separately measured *s*-value of 6.73 S, partial-specific volume of 0.73 mL/g, *dn*/*dc* = 0.185 mL/g, in the without any corrections (black dashed line). Better fits are achieved after accounting for the possible presence of traces of a 10 MDa aggregate (blue, best-fit concentration of 0.07%); additionally considering 5.7 % irreversible dimer that was observed separately in dilute solution (dash-dotted green); and adjusting the constant for scattering contrast by 4% (red solid).

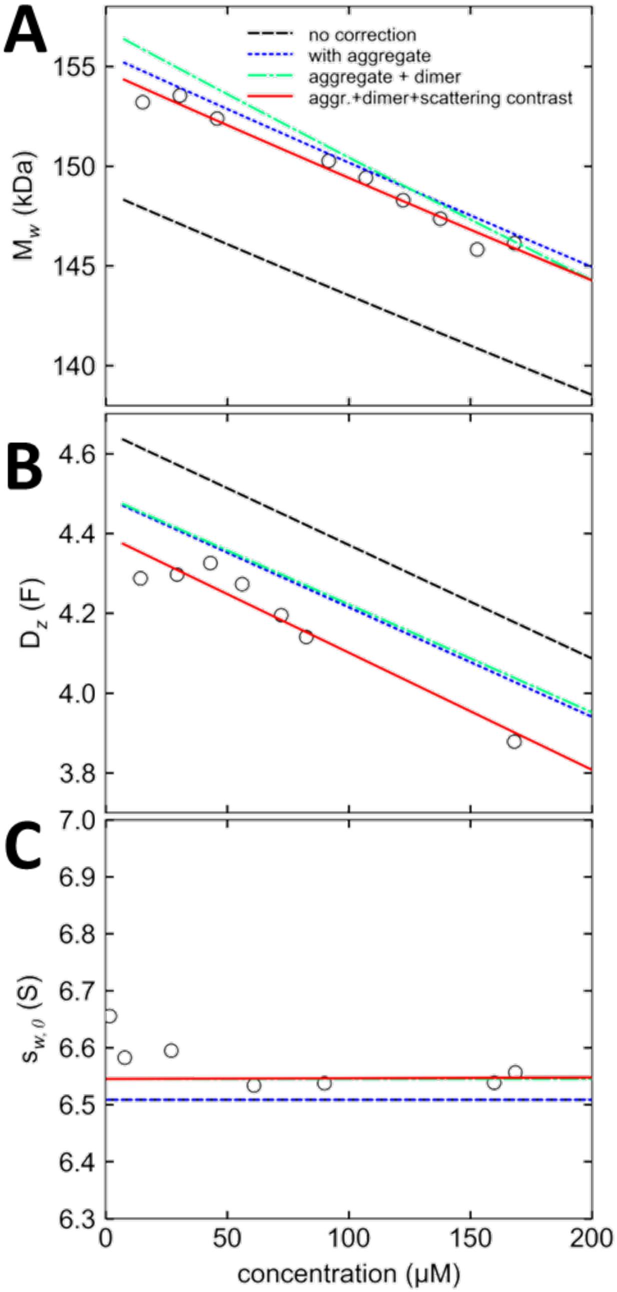

Supplementary Table S1:

Comparison of published interaction parameters

Published best-fit parameters for interaction parameters of different antibodies in PBS, measured with different techniques. Numbers are best-fit interpretations, restricted to reversible interactions.

mAb	ref	K_D* (µM)	B_2 (mL/g)	k_D (mL/g)	k_S (mL/g)
A	[14]	∞ (SV)	0.2 (SV)	-5.2 (DLS)	5.2 (SV)
A	[24]	13,000 (1-2 SV)	2.0 (SV)	1.0 (SV)	5.0 (SV)
**A**	**here**	**7100 (1-2 GMMA)**	**-2.1**	**-9.3**	**5.0**
B	[14]	∞ (SV)	3.8 (SV)	-6.3 (DLS)	8.8 (SV)
B	[24]	∞ (SV)	1.9 (SV)	2.8 (SV)	6.5 (SV)
**B**	**here**	∞	**1.1**	**-4.2**	**6.5**
C	[12]	30/167 (1-3-6 SE)<br>43/148 (1-3-iso LS)	 1.1 (LS)	-59.6 (DLS)	
C	[14]	33 (1-2-iso SV)<br>21 (1-2-iso SE)	8.1 (SV)<br>2.3 (SE)	-64.4 (DLS)	35.4 (SV)
C	[24]	39/33 (1-2-4 SV)	1.8 (SV)	0.1 (SV)	3.6 (SV)
**C**	**here**	**15/48 (1-2-4 GMMA)**	**-5.9**	**-15.4**	**3.6**
D	[14]	604 (1-2, SV)<br>431 (1-2 SE)	19.6 (SV)<br>0.0 (SE)	-10.1 (DLS)	5.6 (SV)
D	[24]	80/1000 (1-2-iso)	-3.0 (SV)	9.5 (SV)	3.5 (SV)
**D**	**here**	**153/5300 (1-2-4-iso GMMA)**	**0.0**	**-3.5**	**3.5**
E	[14]	1029 (1-2 SV)<br>393 (1-2 SE)	10.3 (SV)<br>0.2 (SE)	-19.4 (DLS)	2.8 (SV)
E	[24]	910 (1-2 SV)	-7.4 (SV)	15 (SV)	3.5 (SV)
**E**	**here**	**550 (1-2 GMMA)**	**-7.7**	**-15.6**	**0.2**

*For comparison of affinities in different models, when association equilibrium constants are published these are converted to effective K_D values according to $K_D = (K_{A,1-n})$^(-1/(n-1)).
